# Prognostic role of elevated mir-24-3p in breast cancer and its association with the metastatic process

**DOI:** 10.18632/oncotarget.24403

**Published:** 2018-02-05

**Authors:** Alireza Khodadadi-Jamayran, Betul Akgol-Oksuz, Yelena Afanasyeva, Adriana Heguy, Marae Thompson, Karina Ray, Ariadna Giro-Perafita, Irma Sánchez, Xifeng Wu, Debu Tripathy, Anne Zeleniuch-Jacquotte, Aristotelis Tsirigos, Francisco J. Esteva

**Affiliations:** ^1^ Applied Bioinformatics Laboratories, NYU School of Medicine, New York, NY, USA; ^2^ Department Bioinformatics and Computational Biology, University of Massachusetts Medical School, Worcester, MA, USA; ^3^ Division of Epidemiology, NYU School of Medicine, New York, NY, USA; ^4^ Department of Pathology, NYU School of Medicine, New York, NY, USA; ^5^ Genome Technology Center, NYU School of Medicine, New York, NY, USA; ^6^ Division of Hematology/Oncology, Perlmutter Cancer Center, NYU Langone Health, New York, NY, USA; ^7^ Department of Epidemiology, UT MD Anderson Cancer Center, Houston, TX, USA; ^8^ Department of Breast Medical Oncology, UT MD Anderson Cancer Center, Houston, TX, USA

**Keywords:** breast cancer, gene expression profiling

## Abstract

MicroRNAs have been shown to play important roles in breast cancer progression and can serve as biomarkers. To assess the prognostic role of a panel of miRNAs in breast cancer, we collected plasma prospectively at the time of initial diagnosis from 1,780 patients with stage I-III breast cancer prior to definitive treatment. We identified plasma from 115 patients who subsequently developed distant metastases and 115 patients without metastatic disease. Both groups were matched by: age at blood collection, year of blood collection, breast cancer subtype, and stage. The median follow up was 3.4 years (range, 1-9 years). We extracted RNA from plasma and analyzed the expression of 800 miRNAs using Nanostring technology. We then assessed the expression of miRNAs in primary and metastatic breast cancer samples from The Cancer Genome Atlas (TCGA). We found that, miR-24-3p was upregulated in patients with metastases, both in plasma and in breast cancer tissues. Patients whose primary tumors expressed high levels of miR-24-3p had a significantly lower survival rate compared to patients with low mir-24-3p levels in the TCGA cohort (n=1,024). RNA-Seq data of the samples with the highest miR-24-3p expression versus those with the lowest miR-24-3p in the TCGA cohort identified a specific gene expression signature for those tumors with high miR-24-3p. Possible target genes for miR-24-3p were predicted based on gene expression and binding site, and their effects on cancer pathways were evaluated. Cancer, breast cancer and proteoglycans were the top three pathways affected by miR-24-3p overexpression.

## INTRODUCTION

Breast cancer is the most common malignancy in women, accounting for 30% of new invasive cancers expected to be diagnosed in the US in 2017 [1]. The most important prognostic factor is the stage at presentation, which is determined by the tumor size, the number of axillary lymph nodes involved and the presence/absence of overt metastases. Although the presence of distant metastases at diagnosis (stage IV) is the most ominous sign, women with stage I-III may also harbor occult metastases. It takes years from the initial transformation events of mammary cells to the detection of imaging/clinical changes that result in a breast cancer diagnosis. During this time cancer cells can spread through the circulation to distant sites. The metastatic process is extremely complex and requires adaption mechanisms by cancer cells including but not limited to migration, invasion, penetration into blood vessels, circulation through the bloodstream, extravasation and expansion in distant organs [2]. However, the regulation of the metastatic process at cellular and molecular levels is not well defined for individual patients. Molecular markers that are commonly used in clinical practice include the estrogen receptor (ER), progesterone receptor (PR), the human epidermal growth factor receptor 2 (HER2), the Ki-67 proliferation marker and multi-gene assays [3–5]. These prognostic and predictive factors are used to estimate the risk of recurrence and the potential benefit associated with systemic therapies, including HER2-targeted therapy, endocrine therapy and chemotherapy [6–8]. Despite improvements in local, regional and systemic therapies for breast cancer, 40,610 women are expected to die from metastatic breast cancer in the US in 2017 [1]. Therefore, there is a need to identify novel prognostic and predictive markers of occult metastases to better individualize adjuvant systemic therapy and develop new therapeutic approaches to eradicate occult metastases in women with early-stage breast cancer [9, 10].

MicroRNAs (miRNAs) are small, noncoding RNA molecules 20-23 nucleotides in length that play key roles in regulation of cell division, differentiation, and death [11]. One of the main functions of miRNAs is to silence gene expression by binding to complementary sequences in the 3′UTR of target mRNAs [12]. Dysregulation of miRNA expression has been linked to carcinogenesis, invasion and metastasis [13]. MicroRNAs can be detected and quantified reliably in plasma and tissue from cancer patients and have potential as biomarkers [14, 15]. Previous studies revealed correlations between miRNA detection and prognosis in breast cancer. However, results have not been consistent or reproducible across studies in part because of the different populations evaluated and the methods used to identify miRNAs. Furthermore, there are limited data regarding molecular mechanisms for single miRNAs or miRNA signatures that may impact prognosis in breast cancer patients.

We conducted a study to determine the prognostic role of circulating plasma miRNAs in patients with early-stage breast cancer. We measured the expression of 800 miRNAs in plasma we collected prospectively at the time of diagnosis, before definitive primary treatment. We detected miRNAs that were expressed at higher levels in patients with stage I-III who subsequently developed metastatic disease. We then evaluated the differential expression of miRNAs in primary breast cancer tissue using The Cancer Genome Atlas (TCGA) database. Both analyses revealed mir-24-3p as a top candidate for further evaluation as a biomarker for breast cancer metastasis.

## RESULTS

### Plasma microRNA transcriptome analysis identifies 29 differentially expressed microRNA in patients with early-stage breast cancer who subsequently developed metastasis

**Figure 1:**
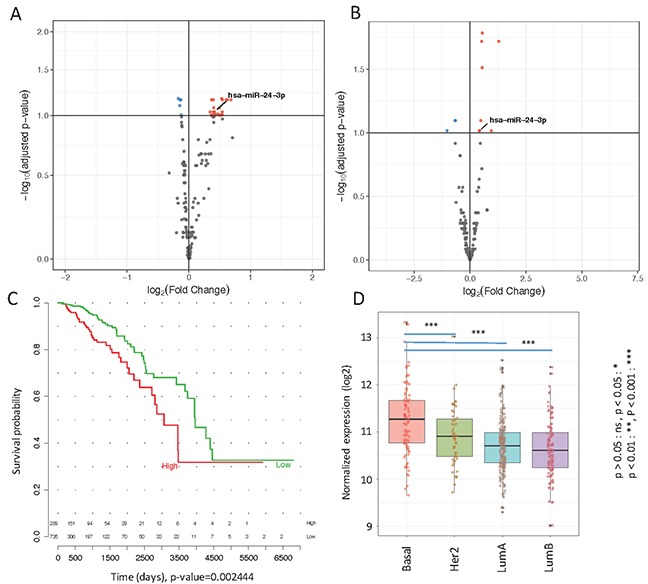
**(A)** Volcano plot of differentially expressed miRNAs in metastatic and non-metastatic serum samples obtained by Nanostring data analysis. Upregulated miRNAs are plotted in red and downregulated in blue **(B)** Volcano plot of differentially expressed miRNAs in TCGA stage I versus stage IV primary tumor samples. Upregulated miRNAs in Stage IV are plotted in red and downregulated in blue **(C)** Survival curve of mir-24-3p in TCGA patients **(D)**. Distribution of mir-24-3p across different tumor subtypes in TCGA primary tumors.

Using Nanostring technology we profiled the microRNA transcriptome in plasma from 115 cases and 115 controls, respectively. In total, we obtained expression levels of ~800 microRNAs. To identify potentially prognostic microRNAs, we performed differential expression analysis between the two patient groups using DEseq2. We identified 29 differentially expressed microRNAs in patients who developed metastasis compared to patients who did not: 24 microRNAs were upregulated while 5 were downregulated (Figure 1A). A comprehensive list of the significantly differentially expressed microRNAs is shown in Table 1.

**Table 1:** List of the significantly differentially expressed microRNAs in plasma of metastatic vs. non-metastatic patients

Gene_name	Control_mean	Metastasis_mean	foldChange	pvalue	padj
hsa-miR-126-3p	421.9134289	676.8186276	1.604164697	0.002945286	0.069244359
hsa-miR-130a-3p	82.35289258	126.8828971	1.540721803	0.003637321	0.069244359
hsa-miR-181a-5p	26.338105	40.11759065	1.523176806	0.001810582	0.069244359
hsa-miR-106a-5p+hsa-miR-17-5p	42.32371603	61.78223478	1.459754496	0.004196628	0.069244359
hsa-miR-15a-5p	57.75558022	84.09167839	1.455992271	0.010034894	0.091986529
hsa-miR-142-3p	307.0759047	447.0948109	1.455974904	0.015969554	0.098567251
hsa-let-7g-5p	85.84623264	124.5787981	1.451185384	0.012432581	0.097684567
hsa-miR-146a-5p	25.87165231	37.48361606	1.448829615	0.000658213	0.067224967
hsa-miR-15b-5p	113.2087794	162.6198738	1.436459917	0.016786562	0.098567251
hsa-miR-199a-5p	34.088818	48.15377871	1.412597489	0.006576129	0.083466246
hsa-miR-191-5p	114.5181748	161.0017431	1.405905598	0.017323941	0.098567251
hsa-miR-27b-3p	17.42951055	24.09008977	1.38214379	0.011079524	0.096216921
hsa-miR-20a-5p+hsa-miR-20b-5p	45.18030217	60.63153392	1.341990447	0.014262422	0.098567251
hsa-let-7d-5p	22.86627562	30.65721483	1.340717454	0.012421539	0.097684567
hsa-miR-24-3p	16.88318331	22.53860549	1.334973689	0.009161539	0.091986529
hsa-miR-19b-3p	27.72094601	36.77714893	1.326691698	0.014876929	0.098567251
hsa-miR-106b-5p	16.56919141	21.93428224	1.323799194	0.006447937	0.083466246
hsa-miR-98-5p	14.76934433	19.54707704	1.323489831	0.009004442	0.091986529
hsa-let-7i-5p	49.96965086	65.92391001	1.31927898	0.008677135	0.091986529
hsa-miR-185-5p	19.81740991	26.10801207	1.317428069	0.002642304	0.069244359
hsa-miR-151a-3p	18.06574804	23.53997512	1.303016906	0.016270605	0.098567251
hsa-miR-92a-3p	18.98911204	24.51292998	1.290893957	0.003233125	0.069244359
hsa-miR-361-5p	14.98231138	19.2511097	1.284922547	0.016185416	0.098567251
hsa-miR-32-5p	21.20346146	26.94874863	1.27095987	0.009784947	0.091986529
hsa-miR-516a-3p+hsa-miR-516b-3p	17.43257585	16.02094928	−1.088111294	0.013722192	0.098567251
hsa-miR-518f-3p	20.49794068	18.74217241	−1.093680083	0.002215359	0.069244359
hsa-miR-125b-5p	19.52071838	17.705905	−1.102497635	0.00400355	0.069244359
hsa-miR-1293	16.4858169	14.89180043	−1.107039876	0.005290175	0.07935262
hsa-miR-1236-3p	17.64853298	15.68462151	−1.125212552	0.000814848	0.067224967

### mir-24-3p overexpression correlates with advanced stage, and triple negative/basal subtype in primary breast cancer tissues

Motivated by the discovery of differentially expressed microRNAs in the plasma of metastatic versus non-metastatic patients, we then hypothesized that some of the microRNA transcripts we detected in the plasma may have originated in the primary tumors either via circulating tumor cell parts, microvesicles or via exosomes. To find supporting evidence for our hypothesis, we analyzed microRNA expression data obtained from TCGA. We then performed differential microRNA expression analysis between stage I and stage IV patients. As demonstrated in the volcano plot in Figure 1B, eleven microRNAs (out of a total of 1881 microRNAs) were significantly upregulated in primary tumors of stage IV versus stage I patients. Interestingly, mir-24-3p was also found to be upregulated in the plasma samples from metastatic versus non-metastatic patients in our cohort. Kaplan-Meier survival analysis shows significantly lower survival rates (p-value=0.0024, quantile-based log-rank test) in patients with elevated levels of mir-24-3p in primary tumors from TCGA (Figure 1C).

For TCGA data, Multivariate Analysis of Variance (MANOVA) indicated the association of mir-24-3p expression with known prognostic factors, i.e. subtype, stage and survival time (p-value= 3.749e-12). Univariate Analysis of Variance (ANOVA and *t*-test) has been performed on tumor subtypes and the results indicate showing a significant (p-value < 0.001) difference between different subtypes (Figure 1D). We also examined the association of mir-24-3p survival with subtype and stage found a positive association in univariate analysis (p = 0.009) that remained statistically significant after adjusting for intrinsic subtype and stage (p = 0.004) (Supplementary Figure 1, right panel (B)). A similar trend was observed in the Nanostring plasma dataset (Supplementary Figure 1, left panel (A)), however it was marginally significant, probably due to the limited number of patients and lower levels of mir-24-3p in plasma compared to tumor tissue. In our patient cohort, there was no difference in mean mir-24-3p between grade I and grade II tumors, but the mean mir-24-3p expression in grade III tumors was higher than in grade I tumors (p=0.04). Grade was not available for TCGA (Supplementary Figure 1).

### mir-24-3p is associated with pathways upregulated in cancer

**Figure 2:**
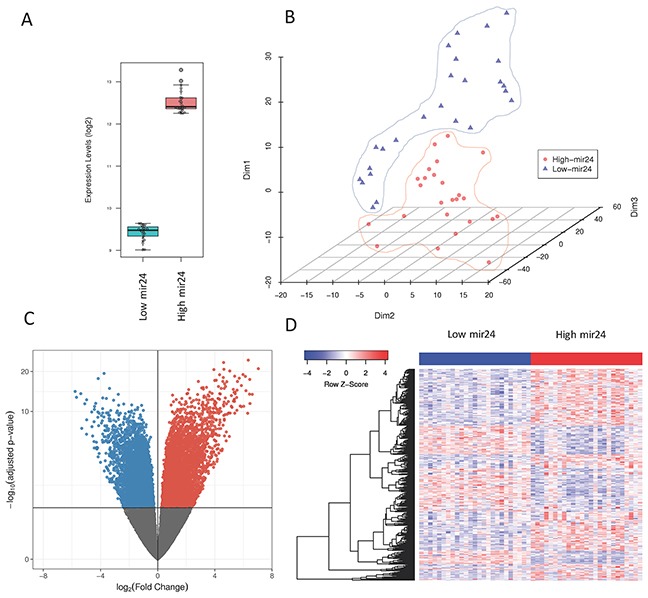
**(A)** Box-plot of mir-24-3p levels in patients showing the highest (n=25) and lowest (n=25) expressing levels of mir-24-3p in primary tumors from the TCGA **(B)** t-SNE plot of patients with high and low mir-24-3p expressing levels showing the separation of the two conditions **(C)** Volcano plot of the genes differentially expressed based on mir-24-3p expression in primary tumors (high versus low) **(D)** Heatmap of the top 500 genes differentially expressed in patients with high versus low mir-24-3p levels.

**Figure 3:**
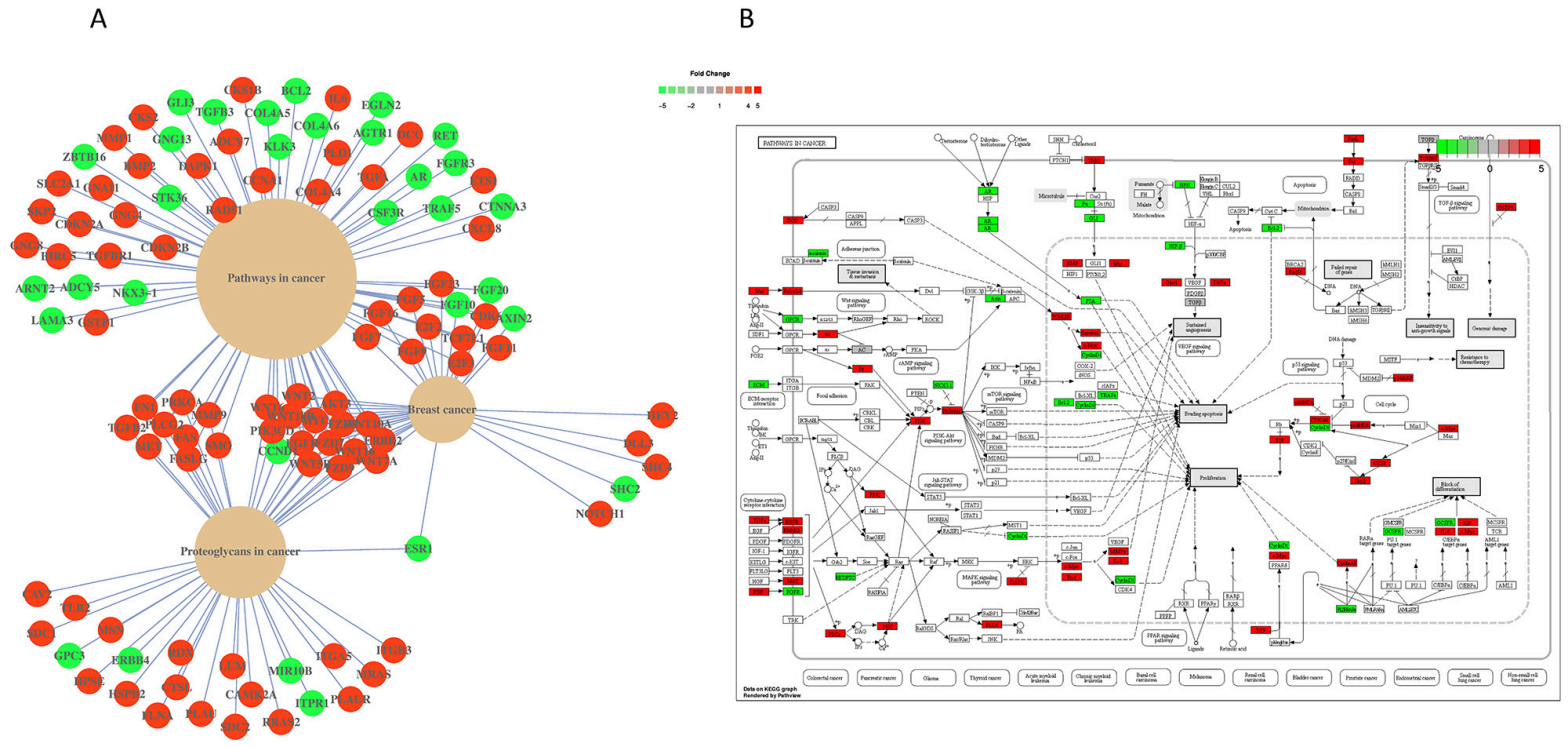
**(A)** The top three significantly (P value < 0.05) effected pathways in patients with high mir-24-3p vs. low mir-24-3p (n=50) **(B)** Heat map overview of the principal pathways involved in cancer (KEGG: hsa05200). Upregulated genes are in red and downregulated genes are shown in green.

Because of the significant correlation of mir-24-3p expression levels with patient survival rates, we further investigated its potential regulatory role in primary tumors. We sorted the TCGA samples based on their mir-24-3p expression and took the top 25 and the bottom 25 samples to perform differential expression analysis on those conditions or two patients groups (Figure 2A). Dimensionality reduction of protein-coding gene expression profiles of the two patient groups using t-distributed stochastic neighbor embedding [16] revealed a good separation of the two patient groups (Figure 2B), suggesting that tumors with high mir-24-3p expression are dependent on transcriptional programs that are distinct from the ones active in patients with low mir-24-3p expression. To identify these transcriptional programs, we first performed differential expression analysis of the protein-coding genes (TCGA RNA-Seq data) between the two patient groups. This analysis yielded a large number of differentially expressed genes (2128 upregulated and 1190 downregulated), highlighted in the volcano plot in Figure 2C. The top 500 significantly differentially expressed genes are depicted using an expression heatmap representation in Figure 2D. Pathway analysis of these protein-coding genes identified several pathways involved in cancer. Top three pathways includes pathways in cancer (KEGG: has05200), proteoglycans in cancer (KEGG: 05205) and breast cancer (KEGG: hsa05224) (Figure 3A). A detailed overview of all the significantly (FDR < 0.1) differentially expressed genes in pathways in cancer (KEGG: has05200 are shown in Figure 3B. Similar pathway overviews were obtained for breast cancer (Supplementary Figure 2) and proteoglycans in cancer (Supplementary Figure 3).

### mir-24-3p protein-coding target genes are involved in cancer pathways

**Figure 4:**
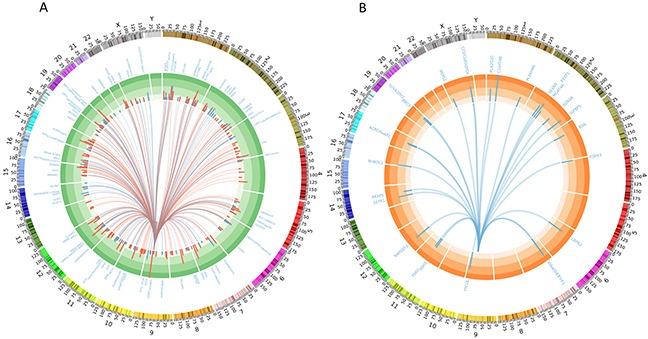
Circos plots depicting **(A)** 316 deferentially expressed genes (FDR < 0.1) out of 745 target genes detected by TargetScan for mir-24-3p (differentially expressed in patients with high mir-24-3p levels vs. low miR-24). Red arcs show genes upregulated and blue downregulated in samples with high mir-24-3p and the histogram represents the fold change. **(B)** 27 significantly down regulated genes (FDR < 0.1 & fold change > 2) out of 745 target genes detected by TargetScan for mir-24-3p showing the most possible targets.

We identified 746 putative protein coding target genes for mir-24-3p using TargetScan (http://www.targetscan.org). We then examined whether mir-24-3p putative targets are significantly differentially expressed in TCGA samples with high mir-24-3p compared to samples with low mir-24-3p expression. Of the 745 genes, 316 genes were significantly differentially expressed (FDR < 0.1). Of these, 134 genes were down-regulated and 182 genes were up-regulated when mir-24-3p levels were high (Figure 4A). To identify the most stringent list of putative targets, we additionally filtered the 316 genes based on fold change and identified 27 genes significantly down-regulated by more than 2-fold in patients with high levels of mir-24-3p (Figure 4B). A pathway analysis of all the target genes revealed that mir-24-3p may significantly regulate genes involved in cancer (p-value < 0.05) (Supplementary Figure 4).

## DISCUSSION

Using nanostring and RNA-sequencing technologies we identified miR-24-3p as a potential novel marker of breast cancer metastases in breast cancer. MiR-24-3p was expressed at high levels in the plasma from early-stage breast cancer patients destined to metastasize despite best available therapy, and in primary breast cancer tissue from patients who presented with stage IV at the time of diagnosis. High expression of mir-24-3p in primary breast cancer tissues correlated with a poor survival rate in TCGA. These data strongly suggest mir-24-3p is involved in the metastatic process in breast cancer patients.

There is great interest in the development of *liquid biopsies* using next generation sequencing. While most studies are focussed on the detection of mutations at the DNA level, Nanostring and RNA-Seq technologies allow detection of nucleic acids in plasma and tissues with high sensitivity and specificity. The demonstration that miRNAs are stable and can be extracted from tissues, blood, and other body fluid without degradation makes them ideal biomarkers for diagnosis and prognosis in breast cancer and other solid tumors. A meta-analysis of studies designed to determine the prognostic role of plasma miRNAs in patients with triple negative breast cancer showed a correlation between high circulating miRNA expression and lower disease-free survival, relapse-free survival and distant metastasis-free survival rates [17]. Sochor and collaborators [18] found that high-risk patients (classified as TNBC, HER2, highly proliferative or with positive node involvement) expressed higher levels in plasma of miRNAs related to cancer (oncomiRs), including miR-2. Several studies have identified mir-24-3p as a potential oncomiR in breast cancer, as its expression is specifically upregulated in both tumor and plasma from patients that developed the disease compare to healthy controls [19].

RNA-Seq data reported by Fiskaa, et al [20] and Hannafon, et al [21] showed mir-24-3p is released from the cells in exosomes. We re-analyzed the data in both studies and found that mir-24-3p is significantly upregulated in breast cancer cell lines versus non-breast cancer cell lines (p-value<0.05 using a t-test). (data not shown).

In addition to the strong correlation between plasma levels of mir-24-3p and the probability of developing metastasis in patients with early-stage of breast cancer, we identified a specific gene expression signature in patients with high levels of mir-24-3p in primary tumors. Interestingly, differential gene expression analysis showed that the principal genes deregulated are affecting the main pathways related to cancer, such as survival, migration and proliferation, pathways directly related to the process of metastasis.

Many studies have linked the expression of mir-24-3p with breast cancer progression and metastasis by regulating genes and signaling pathways associated with cell cycle progression [19], DNA repair and drug resistance [22]. mir-24-3p directly targets p27(Kip1) and p16(Ink4a) in primary keratinocyte and in different cancer derived cell lines promoting their proliferation, suggesting that mir-24-3p is involved in carcinogenesis by post-transcriptional regulation of cyclin-dependent kinase [23]. Du *et al*. observed that mir-24-3p was upregulated in breast cancer tissues compared to benign tissues in a small cohort of patients. *In vitro* and *in vivo* studies indicated that the expression of mir-24-3p enhanced tumor growth, invasion into local tissues, metastasis to lung tissues and decreased overall mouse survival by direct targeting PTPN0 and PTPRF and therefore downregulating phosphorylation levels of EGFR [24]. In another study, the authors reported that mir-24-3p expression was significantly increased in HCC metastatic tumor tissues compared with matched non-metastatic tumor tissues. These authors showed that mir-24-3p could down-regulate p53 through binding to the 3′-UTR of p53 mRNA, resulting in enhanced invasion in HCC cell lines [25]. Additionally, a recent report provides compelling data in support for a direct role of mir-24-3p in promoting breast tumor cell growth and metastasis in a xenograft mouse model via the regulation of ING5 [26]. Altogether, our results suggest that the overexpression of mir-24-3p during early-stages of breast cancer could drive gene-expression reprogramming to a more metastatic phenotype in breast cancer, and that the levels in plasma of mir-24-3p could be used as a feasible biomarker for patient prognosis and therefore help decision-making for treatment options.

In summary, we have identified mir-24-3p as a potential plasma biomarker of occult metastasis in patients with stage I-III breast cancer. Furthermore, we found that mir-24-3p is highly expressed in metastatic breast cancer tissue compared to primary breast cancer tissue, and that those tumors show specific gene expression signature. Our data show mir-24-3p plays an important role in regulation of the metastatic process in breast cancer, and justifies prospective studies to confirm the role of mir-24-3p and its gene targets as novel prognostic and predictive markers.

## MATERIALS AND METHODS

### Study subjects

A whole blood sample was collected prospectively at The University of Texas MD Anderson Cancer Center from 1,780 patients with stage I-III breast cancer, from 6/18/03 to 2/20/2012 after obtaining Institutional Review Board approval and written informed consent from participants. The blood sample was collected after initial diagnosis and prior to definitive treatment, which included either primary surgery or neoadjuvant chemotherapy depending on the clinical stage. All patients were treated uniformly with standard local and systemic therapies. Blood samples were centrifuged at x1200 *g* for 10 minutes at 4°C to separate the blood cells, and the supernatant was transferred into microcentrifuge tubes and then centrifuged a second time at x12,000 *g* for 10 minutes at 4°C to completely remove the cellular components. Plasma was aliquoted and stored at −80°C until use. Blood samples were processed and plasma was frozen within 4 hours of collection. Only patients recruited from 2003 to 2009 were used in the present study to ensure sufficient follow-up. At a median follow up of 3.4 years (range, 1-9 years), 119 patients developed distant metastases. Each of these patients was matched to a patient who was alive and not diagnosed with metastases at follow-up time equal or greater to that at which the case was diagnosed with her first metastasis (control). The matching factors were age (+/− 5 years) at blood collection, year of blood collection, breast cancer subtype, and stage. Out of total 119 cases, 4 cases were excluded because no eligible controls were found. As a result, 115 cases and 115 matched controls are included in the analysis. A summary of the patient characteristics is shown in Table 2.

**Table 2:** Patient characteristics

	Cases n = 115	Controls n = 115
Age at diagnosis, years		
Median (range)	48 (22, 83)	48 (24, 88)
Race/ethnicity		
Caucasian	66 (57%)	69 (60%)
African-American	25 (22%)	16 (14%)
Asian/Pacific Islanders	5 (4%)	6 (5%)
Hispanic	18 (16%)	23 (20%)
Other	1 (1%)	1 (1%)
Menopausal status at diagnosis		
Premenopausal	54 (47%)	58 (51%)
Perimenopausal	4 (3%)	9 (8%)
Postmenopausal	57 (50%)	48 (41%)
Stage at sample collection		
I	4 (3%)	4 (3%)
II	49 (43%)	55 (48%)
III	62 (54%)	56 (49%)
Subtype based on ER, PR, HER2		
ER/PR+ and HER2−	57 (50%)	58 (50%)
HER2+	19 (16%)	18 (16%)
Triple negative	39 (34%)	39 (34%)
Year of sampling		
2003	12 (10%)	8 (7%)
2004	14 (12%)	22 (19%)
2005	23 (20%)	17 (15%)
2006	16 (14%)	14 (12%)
2007	12 (10%)	17 (15%)
2008	27 (23%)	27 (23%)
2009	11 (10%)	10 (9%)
Time to metastases, years		
Median (range)	2.3 (0.6, 9.0)	-

### RNA extraction

Total RNA was extracted from plasma using the Plasma/Serum RNA Purification Midi Kit (Norgen Biotek Corp.) according to the manufacturer’s instructions. Spike in oligos were added as an internal control following the final lysis step according to the manufacturer’s instructions. Eluted RNA volume was brought up to 420μl using nuclease free water and passed through a pre-wet Amicon Ultra-0.5 Centrifugal Filter Device (EMD Millipore). Diluted RNA was spun for 80 minutes at 14,000xg at room temperature and then for 2 minutes at 8,000xg to collect concentrated RNA, at a final volume of 20-25μl.

### Nanostring for assessment of microRNA expression

RNA quality and quantity were analyzed on an agilent Bioanalyzer 2100 using a pico chip. miRNA samples were hybridized using the Nanostring nCounter ® Human v3 miRNA Expression Assay, which includes 800 microRNAs, according to the manufacturer’s protocol. 3uL of concentrated miRNA were used per sample. Hybridizations were processed on the nCounter Prep Station, and prepped cartridges were read on the Nanostring Digital Analyzer using 280 field of view counts. Data was analyzed with Nanostring nSolver 2.0. All samples were analyzed together using the default quality control measures and normalized to spike-ins added to samples before extraction.

### Computational analysis

Raw nanostring data is preprocessed using Nanostring nSolver 2.0. First, the background threshold is determined, either in probe specific or more global manner. Then, the background is subtracted to determine the true counts. This subtraction generally improves downstream data analysis, such as fold-change estimation. The background subtracted nanostring data is then corrected for library sizes and differential expression analysis is performed using DESeq2. The read count tables for all the BRCA miRNA illumine sequencing samples (1207 samples) in TCGA were downloaded from The National Cancer Institute’s (NCI) Genomic Data Commons (GDC). Of these samples, primary solid tumor samples (N=1047, with complete clinical data annotation for cancer stage) and normal tissue samples (N=104) were used in this study. All the read count tables were then corrected for their library size differences based on their geometric library size factors using the DESeq2 package (v3.0) [27]; differential expression (DE) analysis was performed using this package. The PCA plots were visualized using ggplot2 package and the R ‘dist’ function was used to calculate the sample distances by setting the method as Euclidean. Euclidian distance heat-maps were visualized using ggplot2’s “heatmap.2” function. mir-24-3p expression were used for survival analysis using Kaplan-Meier curves (n=1024). Log-rank test was used to test for the survival distributions amongst our sample groups. The mean of miR24 expression was used as a threshold for low and high limits and the survival objects were calculated using the rms R package. Pathway analysis was performed using the clusterprofiler package [28]. We used TargetScan for miRNA target prediction. The Circo plots were generated using CIRCOS [29].

### Statistical analysis

For both data sets, wald test p values and adjusted p values provided by DESeq2 package were used for differential expression analysis (adjusted p < 0.1) which is based on estimating dispersions and uses a negative binomial generalized linear model. For sample clustering, we performed a classical multidimensional scaling or Principal Component Analysis (PCA), t-distributed stochastic neighbor embedding (t-SNE) and a Euclidean distance based clustering. We used ANOVA and MANOVA to examine the association of mir-24-3p with known prognostic factors, i.e. subtype, grade, stage and survival time. For pathway and enrichment analysis, we used hypergeometric distribution tests performed by clusterProfiler package (adjusted p < 0.1). The data in both data sets were individually and comprehensively analyzed and visualized all in the R statistical environment (v3.2.5). The Cox proportional hazards model was used to assess the association of subtype, stage, and mir-24-3p with survival in the TCGA dataset. The log-rank test was also used for testing the survival rates and distributions.

### Statement of significance

We identified mir-24-3p as a novel marker of occult metastases in plasma from patients with stage I-III breast cancer using nanostring technology. RNA-Seq data from The Cancer Genome Atlas showed association of mir-24-3p expression and metastases in primary breast cancer tissue. Expression of mir-24-3p was associated with poor survival, suggesting a potential role as a novel prognostic marker in breast cancer. Furthermore, molecular pathways regulated by mir-24-3p are potential therapeutic targets for metastatic breast cancer.

### Data accession

All the raw count tables for nanostring data and it’s quality control tests areprovided in the supplementary data (Supplementary data 2 and 3). Theclinical metadata for the nanostring samples are provided in supplementary data 1. TCGA mir-24-3p expression profiles and their clinical information areprovided in supplementary data 4.

## SUPPLEMENTARY MATERIALS FIGURES AND DATAS










