# Supplementary material for: Prognostic role of elevated mir-24-3p in breast cancer and its association with the metastatic process
Source: Oncotarget. 2018 Feb 5;9(16):12868–78. doi: 10.18632/oncotarget.24403 (PMC5849180; doi:10.18632/oncotarget.24403)
Supplement: Supplementary file 1 [file oncotarget-09-12868-s001.pdf]

Prognostic role of elevated mir-24-3p in breast cancer and its association with the metastatic process

SUPPLEMENTARY MATERIALS

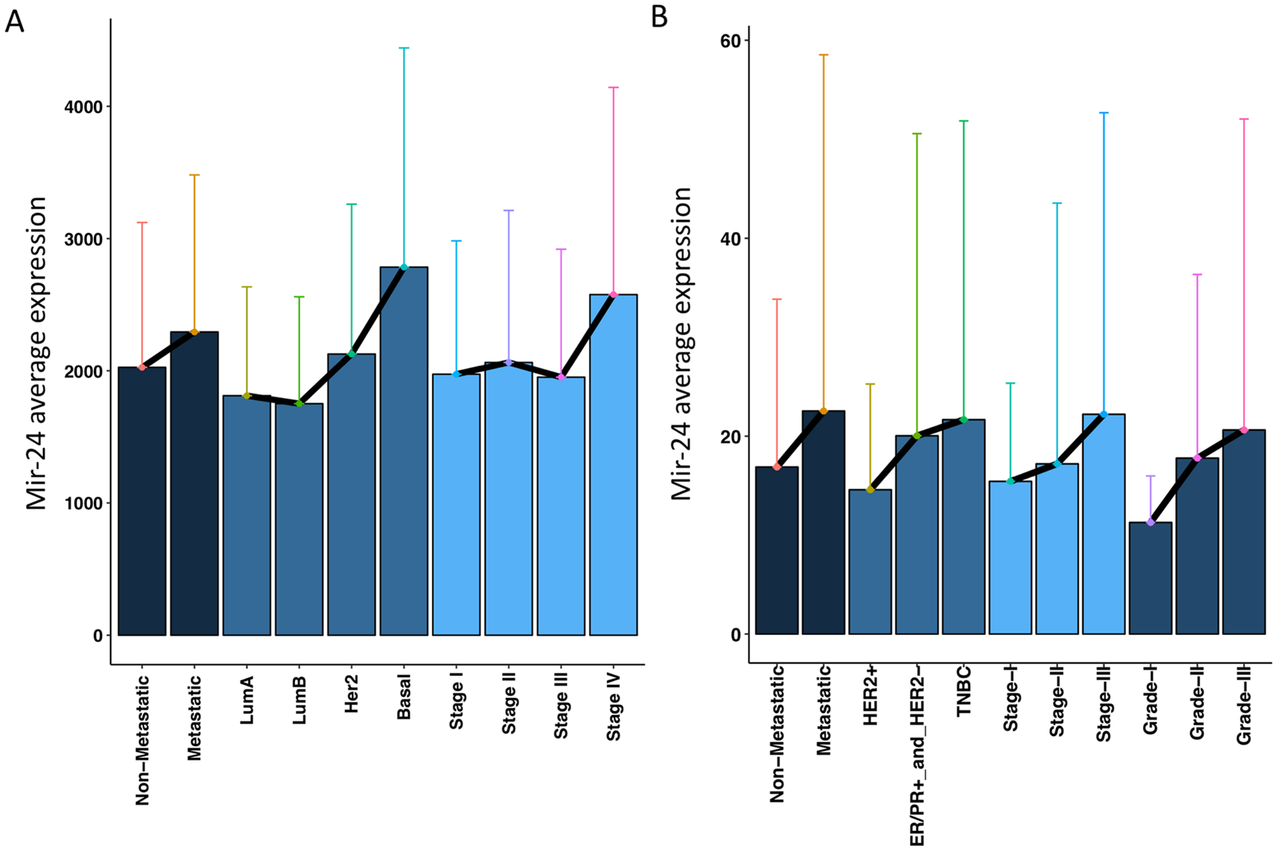

Supplementary Figure 1: (A) Mean bar plot for Nanostring data, showing metastasis status, subtype, stage and grade. **(B)** Mean bar plot for TCGA data showing metastasis status, subtype and stage.

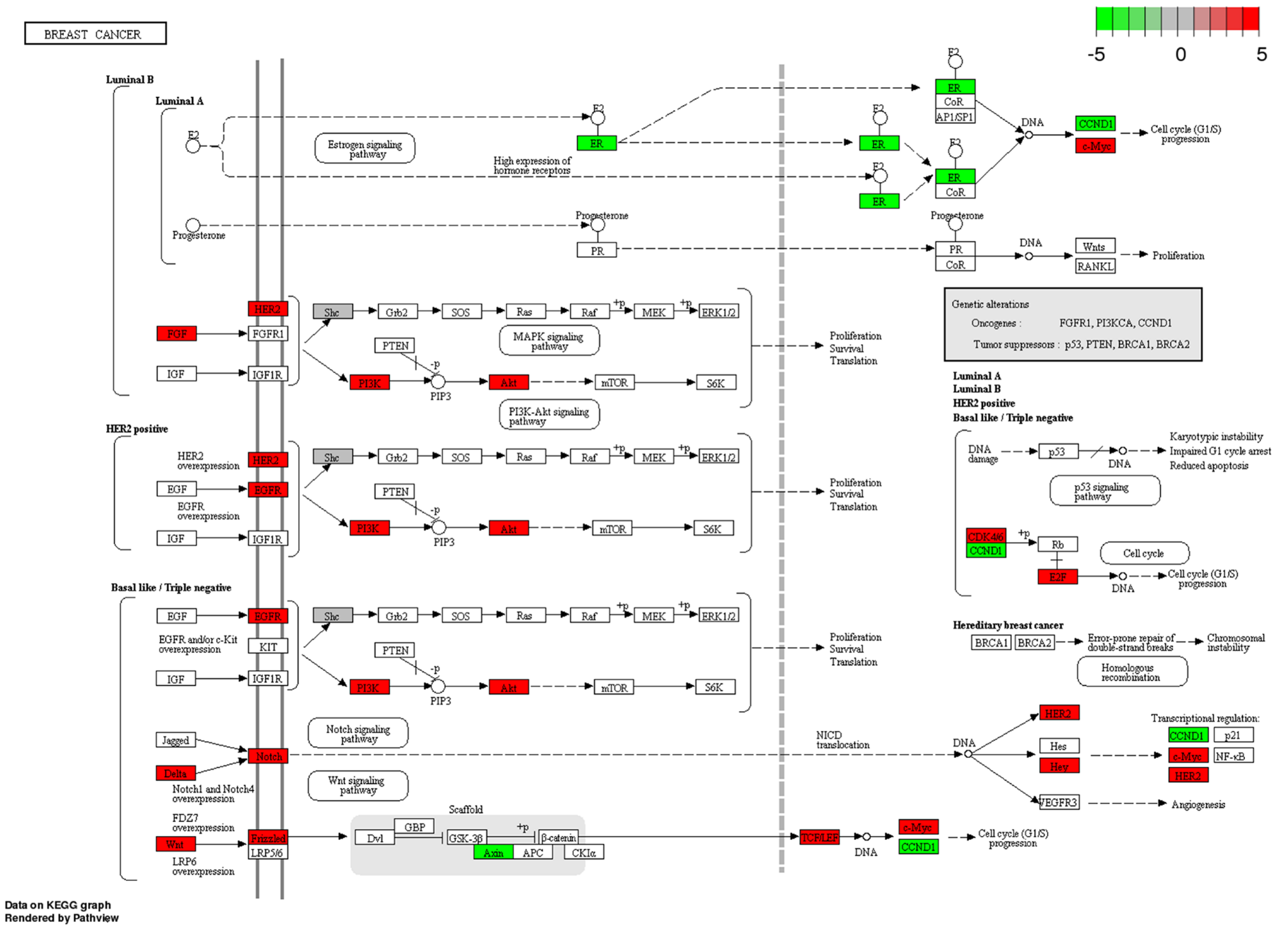

Supplementary Figure 2: Heatmap overview of breast cancer pathway (KEGG: hsa05224). Upregulated genes are in red and downregulated genes are shown in green.

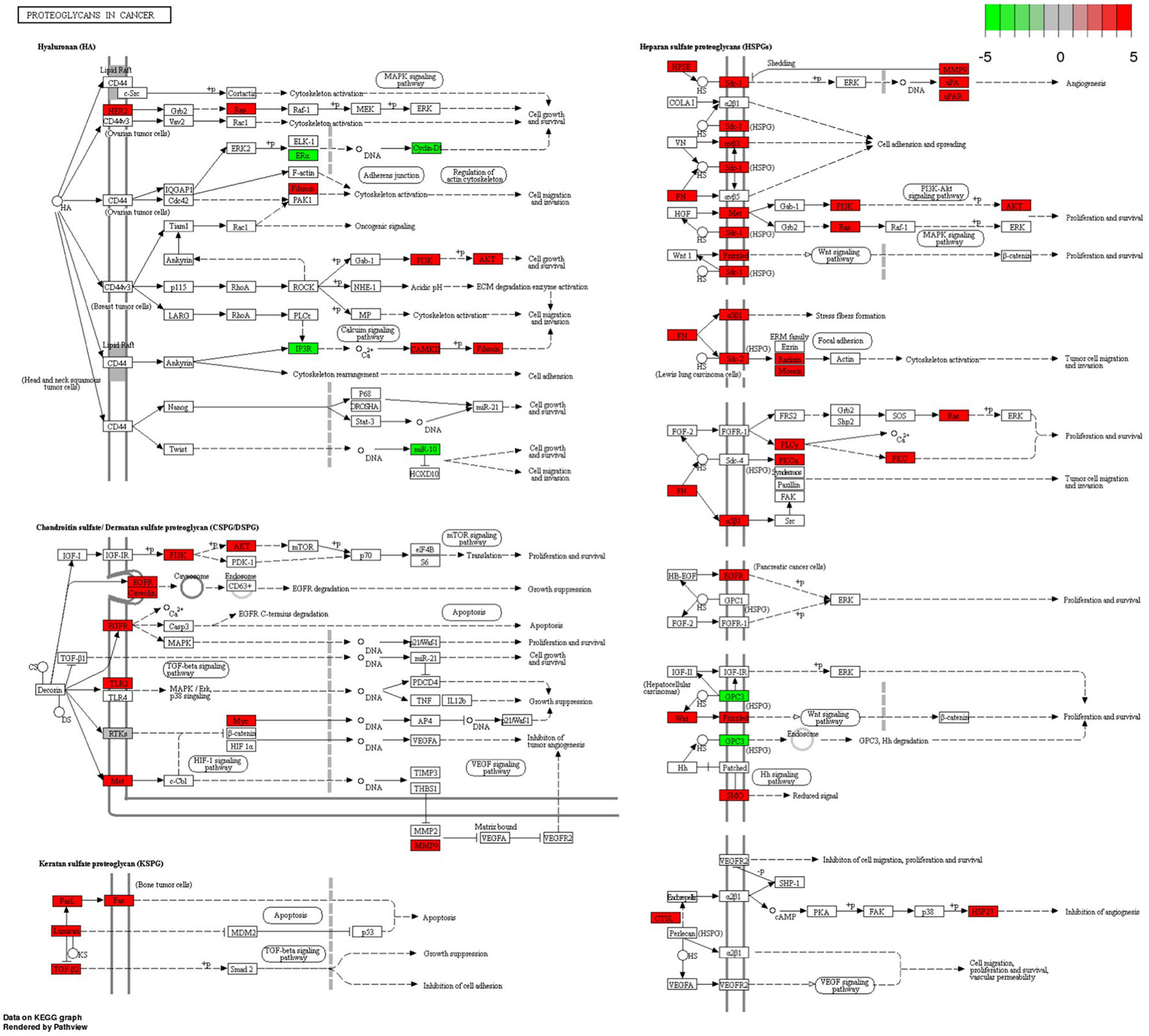

Supplementary Figure 3: Heatmap of proteoglycans in cancer (KEGG: hsa05205). Upregulated genes are in red and downregulated genes are shown in green.

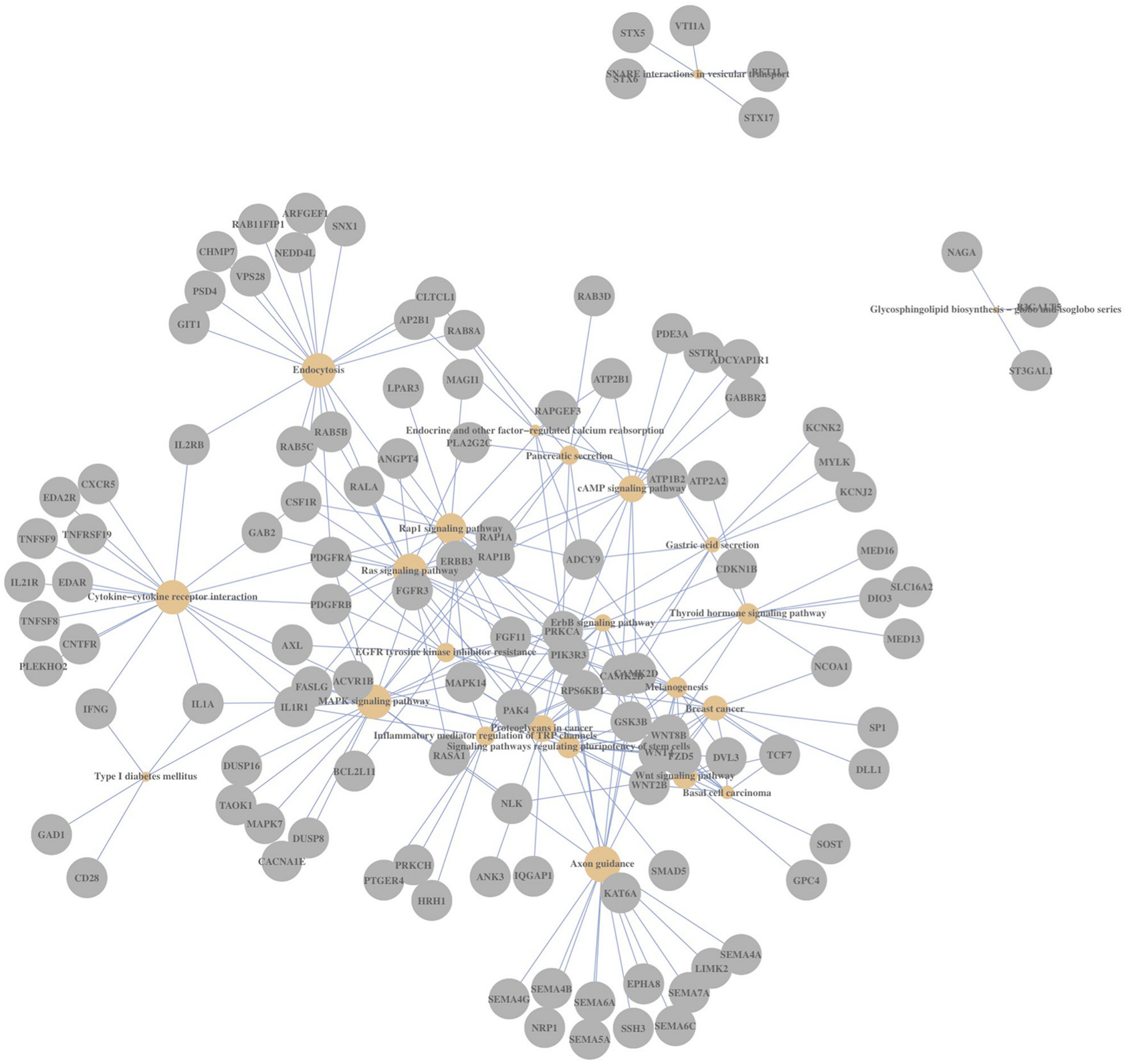

Supplementary Figure 4: Significantly (P value < 0.05) effected pathways for mir-24-3p target genes detected by TargetScan.

Supplementary Data 1: Nanostring clinical and normalized mir-24 data.

See Supplementary File 1

Supplementary Data 2: Nanostring raw data.

See Supplementary File 2

Supplementary Data 3: Nanostring QC data.

See Supplementary File 3

Supplementary Data 4: TCGA clinical and normalized mir-24 data.

See Supplementary File 4
