# Supplementary material for: Prognostic role of elevated mir-24-3p in breast cancer and its association with the metastatic process
Source: Oncotarget. 2018 Feb 5;9(16):12868–78. doi: 10.18632/oncotarget.24403 (PMC5849180; doi:10.18632/oncotarget.24403)
Supplement: Supplementary file 4 [file oncotarget-09-12868-s004.pdf]

Assay Class:	Eukaryote Total RNA Pico	Created:	5/4/2015 12:48:11 PM
Data Path:	C:\...Eukaryote Total RNA Pico_DE72903144_2015-05-04_12-48-12.xad	Modified:	9/13/2017 3:37:51 PM

Electropherogram Summary

Ladder

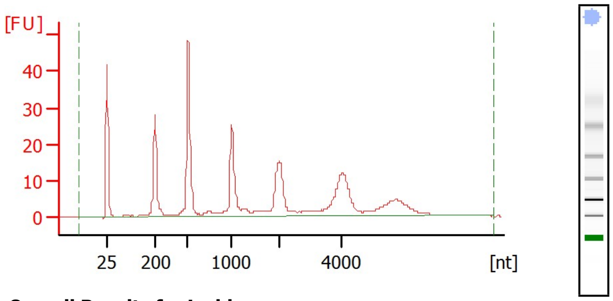

Overall Results for Ladder

RNA Area:	336.6
RNA Concentration:	1,000 pg/µl
Result Flagging Color:	
Result Flagging Label:	All Other Samples

9a

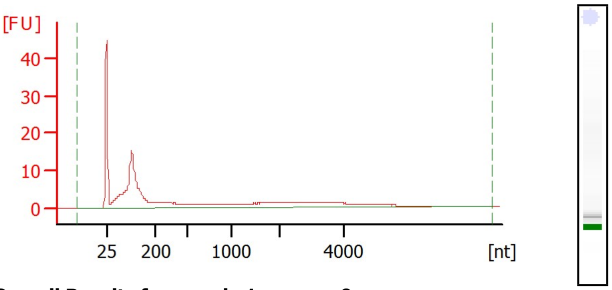

Overall Results for sample 1 : 9a

RNA Area:	159.4
RNA Concentration:	474 pg/µl
rRNA Ratio [28s / 18s]:	0.0
RNA Integrity Number (RIN):	1.9 (B.02.08)
Result Flagging Color:	
Result Flagging Label:	RIN: 1.90

9b

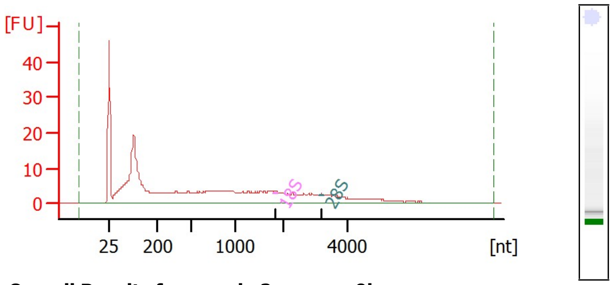

Overall Results for sample 2 : 9b

RNA Area:	298.3
RNA Concentration:	886 pg/µl
rRNA Ratio [28s / 18s]:	0.5
RNA Integrity Number (RIN):	2.4 (B.02.08)
Result Flagging Color:	
Result Flagging Label:	RIN: 2.40

Fragment table for sample 2 : 9b

Name	Start Size [nt]	End Size [nt]	Area	% of total Area
18S	1,803	1,899	0.2	0.1
28S	3,146	3,251	0.1	0.0

9c

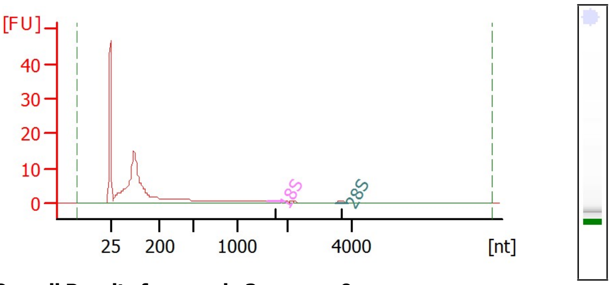

Overall Results for sample 3 : 9c

RNA Area:	131.8
RNA Concentration:	392 pg/µl
rRNA Ratio [28s / 18s]:	0.7
RNA Integrity Number (RIN):	2.4 (B.02.08)
Result Flagging Color:	
Result Flagging Label:	RIN: 2.40

Fragment table for sample 3 : 9c

Name	Start Size [nt]	End Size [nt]	Area	% of total Area
18S	1,622	1,950	0.7	0.6
28S	3,525	3,848	0.5	0.4

 © Copyright 2003 - 2015 Agilent Technologies, Inc.

Assay Class: Eukaryote Total RNA Pico
Data Path: C:\...Eukaryote Total RNA Pico_DE72903144_2015-05-04_12-48-12.xad

Created: 5/4/2015 12:48:11 PM
Modified: 9/13/2017 3:37:51 PM

Electropherogram Summary Continued ...

9d

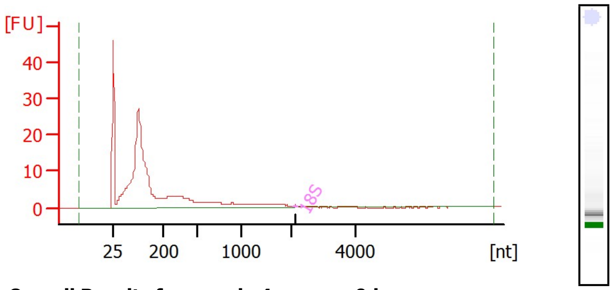

Overall Results for sample 4 : 9d

RNA Area: 230.4
RNA Concentration: 685 pg/µl
rRNA Ratio [28s / 18s]: 0.0
RNA Integrity Number (RIN): 2.5 (B.02.08)
Result Flagging Color:
Result Flagging Label: RIN: 2.50

Fragment table for sample 4 : 9d

Name	Start Size [nt]	End Size [nt]	Area	% of total Area
18S	2,096	2,199	0.2	0.1

9e

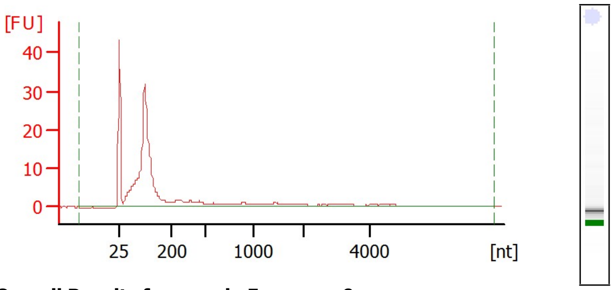

Overall Results for sample 5 : 9e

RNA Area: 207.7
RNA Concentration: 617 pg/µl
rRNA Ratio [28s / 18s]: 0.0
RNA Integrity Number (RIN): 2.5 (B.02.08)
Result Flagging Color:
Result Flagging Label: RIN: 2.50

9f

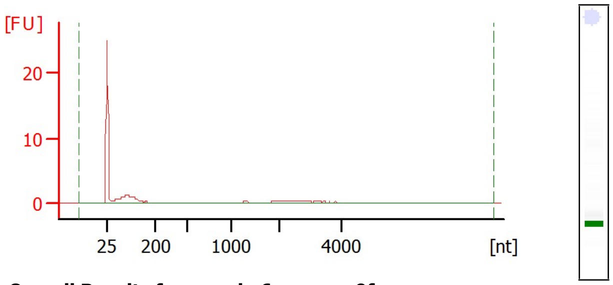

Overall Results for sample 6 : 9f

RNA Area: 19.4
RNA Concentration: 58 pg/µl
rRNA Ratio [28s / 18s]: 0.0
RNA Integrity Number (RIN): 1 (B.02.08)
Result Flagging Color:
Result Flagging Label: RIN:1

9g

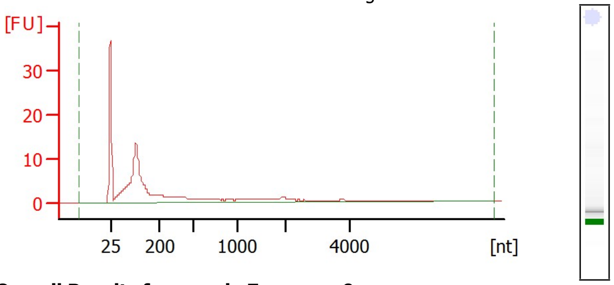

Overall Results for sample 7 : 9g

RNA Area: 125.7
RNA Concentration: 374 pg/µl
rRNA Ratio [28s / 18s]: 0.0
RNA Integrity Number (RIN): 2.4 (B.02.08)
Result Flagging Color:
Result Flagging Label: RIN: 2.40

 © Copyright 2003 - 2015 Agilent Technologies, Inc.

Assay Class: Eukaryote Total RNA Pico
Data Path: C:\...Eukaryote Total RNA Pico_DE72903144_2015-05-04_12-48-12.xad

Created: 5/4/2015 12:48:11 PM
Modified: 9/13/2017 3:37:51 PM

Electropherogram Summary Continued ...

9h

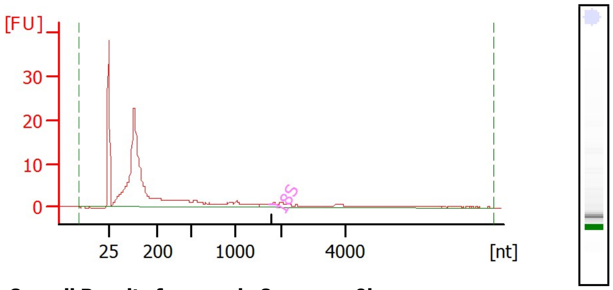

Overall Results for sample 8 : 9h

RNA Area: 180.0
RNA Concentration: 535 pg/µl
rRNA Ratio [28s / 18s]: 0.0
RNA Integrity Number (RIN): 2.5 (B.02.08)
Result Flagging Color:
Result Flagging Label: RIN: 2.50

Fragment table for sample 8 : 9h

Name	Start Size [nt]	End Size [nt]	Area	% of total Area
18S	1,722	1,863	0.5	0.3

9i

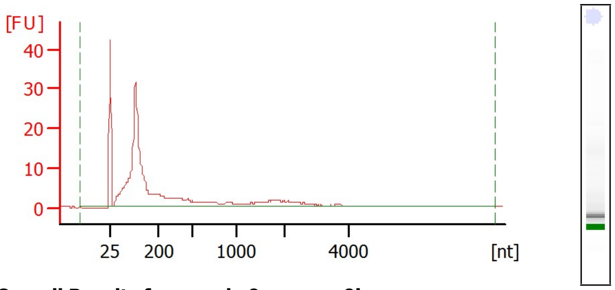

Overall Results for sample 9 : 9i

RNA Area: 231.8
RNA Concentration: 689 pg/µl
rRNA Ratio [28s / 18s]: 0.0
RNA Integrity Number (RIN): 2.5 (B.02.08)
Result Flagging Color:
Result Flagging Label: RIN: 2.50

9j

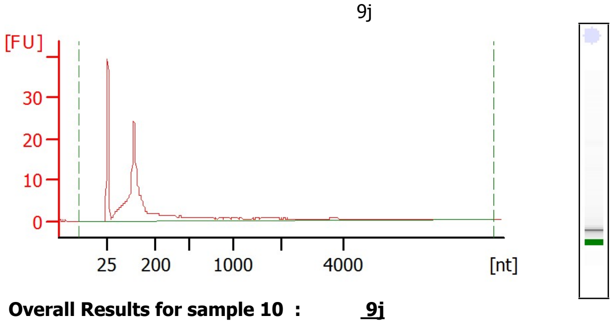

Overall Results for sample 10 : 9j

RNA Area: 147.3
RNA Concentration: 438 pg/µl
rRNA Ratio [28s / 18s]: 0.0
RNA Integrity Number (RIN): 2.5 (B.02.08)
Result Flagging Color:
Result Flagging Label: RIN: 2.50

9k

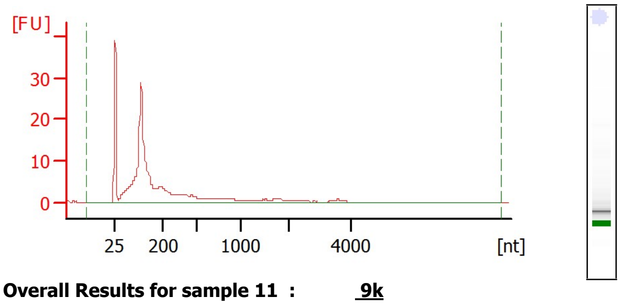

Overall Results for sample 11 : 9k

RNA Area: 183.7
RNA Concentration: 546 pg/µl
rRNA Ratio [28s / 18s]: 0.0
RNA Integrity Number (RIN): 2.5 (B.02.08)
Result Flagging Color:
Result Flagging Label: RIN: 2.50

 © Copyright 2003 - 2015 Agilent Technologies, Inc.
